# Radical Prostatectomy Combined with Prostate Specific Membrane Antigen–radioguided Lymph Node Dissection is Associated with Longer Treatment-free Survival for Patients with Primary Lymph Node–positive Prostate Cancer

**DOI:** 10.1016/j.euros.2025.10.018

**Published:** 2025-11-14

**Authors:** Philipp Korn, Flemming Lischewski, Helena Staehler, Matthias Eiber, Tobias Maurer, Thomas Horn, Jürgen E. Gschwend, Matthias M. Heck

**Affiliations:** a Department of Urology, Rechts der Isar Medical Center, Technical University of Munich, Munich, Germany; b Department of Nuclear Medicine, Rechts der Isar Medical Center, Technical University of Munich, Munich, Germany; c Department of Urology and Martini-Klinik Prostate Cancer Center, University Hospital Hamburg-Eppendorf, Hamburg, Germany; d Department of Urology, University of Augsburg, Augsburg, Germany

**Keywords:** High-risk prostate cancer, Lymph node metastases, Metastatic hormone-sensitive prostate cancer, Prostate-specific membrane antigen, Positron emission tomography, Radical prostatectomy, Radioguided surgery, Salvage therapy

## Abstract

**Background and objective:**

The optimal treatment for patients with prostate cancer with primary lymph node metastases (LNMs) is still a matter of debate. Radical prostatectomy (RP) combined with prostate-specific membrane antigen (PSMA)-radioguided surgery (RGS) may be a helpful technique in removal of LNMs detected on preoperative PSMA positron emission tomography (PET) in comparison to conventional lymph node dissection (LND). The aim of our retrospective analysis was to determine whether addition of PSMA-RGS at primary radical prostatectomy (RP) is associated with longer survival.

**Methods:**

^99m^Technetium-PSMA-I&S was administered preoperatively to facilitate LND during RP in the RGS group. Standard descriptive statistics were used to outline differences in patient characteristics. To address imbalance of covariates, we created matched samples using exact matching before assessing survival outcomes using Cox regression analyses.

**Key findings and limitations:**

Matched samples were created for a cohort comprising 46 patients who underwent RP with RGS, and 42 patients who underwent RP without RGS, with matching according to LNM distribution, number of LNMs on preoperative PSMA PET scans, and surgical margin status. Multivariate Cox regression revealed an association between longer treatment-free survival (TFS) and PSMA RGS (hazard ratio [HR] 0.53, 95% confidence interval [CI] 0.30–0.94). A higher number of positive lymph nodes (HR 1.29, 95% CI 1.07–1.56) and positive surgical margins (HR 1.86, 95% CI 1.06–3.25) were associated with shorter TFS. The main limitations are the retrospective design and small sample size.

**Conclusions and clinical implications:**

Addition of PSMA-RGS at primary RP was associated with longer TFS for patients with limited PSMA PET–positive locoregional LNMs.

**Patient summary:**

We looked at whether a technique to label and detect lymph node metastases during prostate cancer surgery is linked to better cancer control for patients whose preoperative scans showed limited spread to the lymph nodes. We found that this approach may lead to better cancer control after surgery and a longer time before additional treatment is needed.

## Take Home Message

Radical prostatectomy combined with prostate-specific membrane antigen (PSMA) radioguided surgery (RGS) for lymph node dissection was associated with a higher detection rate for lymph node metastases and higher probability of reaching prostate-specific antigen <0.07 ng/ml without further treatment. In addition, PSMA-RGS was associated with longer treatment-free survival in a highly selected patient cohort.

## 1 Introduction

Lymph node metastases (LNMs) are common among patients with high-risk prostate cancer. Therefore, preoperative staging is recommended in current guidelines [1]. Prostate-specific membrane antigen (PSMA) positron emission tomography (PET) has higher accuracy than conventional imaging for staging in terms of LNM localization and extent [[2], [3], [4]].

There are limited data on the optimal treatment for patients with LNMs in prostate cancer, especially for those diagnosed before any planned treatment (cN1). For curative-intent surgery, removal of all LNMs is generally considered important, but can be challenging because of the small size and unusual location of LNMs. In this setting, PSMA-radioguided surgery (PSMA-RGS) may be a helpful theranostic technique to facilitate LNM removal. PSMA-RGS was initially established in 2014 [5]. Since then, retrospective data from large cohorts of more than 500 patients have shown higher successful rates for LNM removal in comparison to conventional salvage lymph node (LN) dissection [6,7]. Moreover, PSMA-RGS achieved a meaningful oncological benefit with regard to postoperative treatment-free survival (TFS), especially in patients with low prostate-specific antigen (PSA) before surgery [7]. In the primary treatment setting, radical prostatectomy (RP) combined with PSMA-RGS has only been recently reported. Three prospective studies with small sample sizes showed that PSMA-RGS is safe and feasible, with per-lesion sensitivity of 63–76% and specificity of 96–99% [[8], [9], [10]]. Our group reported retrospective data for 35 patients with LNMs on preoperative PSMA PET who underwent RP with PSMA-RGS, with LNMs successfully removed in 94% of cases [11]. However, the oncological benefit of PSMA-RGS at primary RP still remains unclear. In this retrospective analysis, we compared postoperative outcomes for patients who underwent RP with pelvic LN dissection (PLND) with or without PSMA-RGS assistance in an adjusted matched case-control study.

## 2 Patients and methods

### 2.1 Study population

Overall, 96 patients underwent open RP for histologically proven prostate cancer with evidence of LNMs on preoperative PSMA PET at our institution between January 2016 and June 2023. We excluded patients with prior hormonal therapy and with LNMs above the aortic bifurcation on PSMA PET. RP was performed with extended PLND (ePLND) with or without PSMA-RGS according to a predefined template that included the iliac vessels (external, internal, and common) and the obturator fossa. All patients provided written informed consent to the procedure and data collection, and in cases undergoing PSMA-RGS, the experimental nature of the surgical method. The retrospective evaluation of patients was conducted in accordance with the criteria of the Helsinki Declaration and was approved by the ethics committee of the Technical University of Munich (permit 30/22 S-NP).

### 2.2 RP procedure with PSMA-RGS

We have previously reported the method for RP with PSMA-RGS [11]. In brief, ^99m^Tc-PSMA-I&S was injected the day before surgery. LNMs were detected intraoperatively using a gamma probe (Crystal Probe CXSSG603; Crystal Photonics, Berlin, Germany) with acoustic feedback. Ex vivo measurements (rated as positive when the signal was at least twice the background signal) were performed to confirm PSMA positivity in the resected tissue [12].

### 2.3 Outcomes of interest

The complete PSA response (defined as PSA <0.07 ng/ml) without any additional treatment was determined in week 4–12 after RP. If multiple assessments were performed (eg, to confirm PSA persistence), the lowest value was used to define PSA response. Biochemical recurrence (BCR) was defined as confirmed PSA >0.2 ng/ml after surgery. If a patient did not experience PSA <0.2 ng/ml after RP (PSA persistence), the date of first PSA measurement after RP was defined as the date of BCR. If a rising PSA triggered treatment before the level reached >0.2 ng/ml, the date of treatment initiation was defined as the date of BCR. BCR-free survival (bRFS) was defined as the time from surgery to the date of BCR.

TFS was defined as the time from surgery to the first date of any salvage therapy, such as salvage radiotherapy (RTx), androgen deprivation therapy (ADT), or repeat PSMA-RGS. Supplementary Fig. 1 shows a flowchart of the study cohort and the outcomes.

### 2.4 Statistical analyses

Standard descriptive methods were used to assess patient characteristics, with a Wilcoxon rank-sum test applied for between-group comparison of continuous variables, and a χ^2^ test for categorical variables. Patients were divided into two groups according to whether ePLND was with or without PSMA-RGS assistance. To address imbalance between the groups, matched samples were created using different matching approaches. The variables used for matching were the number of positive LNs on preoperative PSMA PET (coded as 0–2, 3–4. or ≥5 positive LNs), surgical margin status, and LN distribution on preoperative PSMA PET scans (whether located within or outside the ePLND field). The final balance was assessed using the standardized mean differences (SMD; <0.2 considered acceptable) for the matched variables, which resulted in selection of the “exact matching” method [13]. Logistic regression was applied to evaluate the association between PSMA-RGS and complete PSA response after surgery. Multivariate Cox regression was used to examine the association of PSMA-RGS with bRFS and TFS, with adjustment for the covariates shown in the respective tables and exclusion of patients with R_X_ status from the model. Because the cohort was created via exact matching with near-equal group sizes, the fitted Cox models were unweighted. Patients without an event were censored at the date of their last disease assessment. For bRFS, this was the date of the last PSA assessment; for TFS, it was the date of the most recent follow-up. Adjusted survival curves with the same covariates as specified for the Cox models were used to estimate survival times and visualize the multivariate Cox regression results. R v3.4.3 with the packages *MatchIt*, *Survival*, and *adjustedCurves* was used for statistical analysis.

## 3 Results

Between January 2016 and June 2023, 96 patients with LNMs on preoperative PSMA PET underwent RP, of whom 53 had RP + ePLND with PSMA-RGS assistance (between June 2018 to June 2023) and 43 had RP + conventional ePLND (between January 2016 and December 2020). Most of the patients who did not have RGS were treated before the introduction of PSMA-RGS at our institution (*n* = 29; 67% of the conventional ePLND group). However, there was an overlap during which both modalities were provided (*n* = 16, 30% of the PSMA-RGS group).We detected imbalances in key variables (number of LNMs on preoperative PSMA PET, surgical margin status, LNMs within vs outside the ePLND field) that might have an impact on analyses. To address these imbalances, we assessed matching methods, including nearest neighbor matching and optimal matching, using a calculated propensity score by analyzing the estimated SMD. Only exact matching resulted in balanced variables. After exact matching, 46 patients from the PSMA-RGS group and 42 from the conventional ePLND group were eligible for further analyses. Patient characteristics after matching are described in Table 1.

**Table 1:** Patient characteristics

	Unmatched cohort			Matched cohort			
	RP + RGS (*n* = 53)	RP without RGS (*n* = 43)	*p* value a	RP + RGS (*n* = 46)	RP without RGS (*n* = 42)	SMD b	*p* value a, b
Median age at surgery, yr (IQR)	67 (64–72)	68 (62–74)	0.8	68 (63–72)	68 (61–74)		0.4
Median initial PSA, ng/ml (IQR)	17.8 (9.2–41.0)	19.7 (7.4–37.5)	0.5	17.4 (9.4–42.3)	20.3 (7.9–37.8)		0.5
Median number of PET-positive nodes (IQR)	2 (1–3)	2 (1–3)	0.2	2 (1–3)	2 (1–3)		0.3
Number of PET-positive nodes, *n* (%)			0.4			0	
0–2 positive nodes	27 (51)	28 (65)		24 (52)	27 (63)		
3–4 positive nodes	19 (44)	11 (26)		15 (33)	11 (26)		
≥5 positive nodes	7 (13)	4 (9)		7 (15)	4 (10)		
Distribution of lymph nodes, *n* (%)			**0.02**			0	
Infield only	29 (55)	34 (79)		29 (63)	33 (79)		
Out of field	24 (45)	9 (21)		17 (37)	9 (21)		
pT stage, *n* (%)			0.8				>0.9
pT2a–c	8 (15)	4 (9)		5 (11)	4 (10)		
pT3a	7 (13)	7 (16)		6 (13)	7 (17)		
pT3b	37 (70)	31(72)		34 (74)	30 (71)		
pT4a	1 (2)	1 (2)		1 (2)	1 (2)		
pN stage, *n* (%)			0.3				0.2
pN0	3 (6)	6(14)		2 (4)	6 (14)		
pN1	50 (94)	37 (86)		44 (96)	36 (86)		
Median number of positive nodes on HPx (IQR)	3 (2–5)	2 (1–3)	**0.04**	4 (2–6)	2 (1–3)		**0.03**
Median number of resected lymph nodes (IQR)	24 (20–33)	26 (20–29)	0.8	24 (20–33)	26 (20–29)		0.8
Gleason score, *n* (%)			**0.04**				0.10
7a + 7b	26 (49)	19 (44)		23 (50)	19 (45)		
8	6 (11)	0 (0)		4 (9)	0 (0)		
9	21 (40)	24 (56)		19 (41)	23 (55)		
R stage, *n* (%)			0.7			0	
R0	25 (47)	18 (42)		18 (39.1)	18 (42.9)		
R1	27(51)	23 (53)		27 (50.9)	23 (54.8)		
Rx	1 (2)	2 (5)		1 (2.2)	1 (2.4)		
Median follow-up, mo (IQR) c							
For biochemical recurrence–free survival	36 (22–47)	65 (23–67)		37 (22–47)	65 (23–67)		
For treatment-free survival	32 (10–37)	69 (46–75)		35 (22–42)	69 (46–75)		

HPx = histopathology; IQR = interquartile range; PET = positron emission tomography; PSA = prostate-specific antigen; RGS = radioguided surgery; RP = radical prostatectomy.

a The *p* values were calculated using a Wilcoxon rank-sum test for continuous variables, and a χ^2^ test for categorical variables.

b Standardized mean difference (SMD) is reported for variables used for matching; *p* values are reported for all other variables.

c Median follow-up times are reported for the group of patients without an event.

LNMs were detected in 44 patients (96%) in the ePLND + PSMA-RGS group and 36 patients (86%) in the conventional ePLND group (*p* = 0.02). After matching, the number of positive nodes on preoperative PSMA PET was the same in the two groups (median 2, interquartile range [IQR] 1–3), whereas the number of LNMs resected was significantly higher in the PSMA-RGS group (median 4, IQR 2–5.5) than in the conventional ePLND group (median 2, IQR 1–3; *p* = 0.03). The LNM count on PSMA PET and final histopathology matched in 19 (22%) patients overall, including nine patients in the PSMA-RGS group (20%) and ten in the conventional ePLND group (21%). PSMA PET underestimated disease extent in 47 patients (53%) overall, including 29 in the PSMA-RGS group (63%) and 18 in the conventional ePLND group (43%). More LNs were detected on preoperative PSMA PET than in the final histopathological specimen in 22 patients (25%) overall, including eight in the PSMA RGS group (17%) and 14 in the conventional ePLND group (33%).

**Fig. 1:**
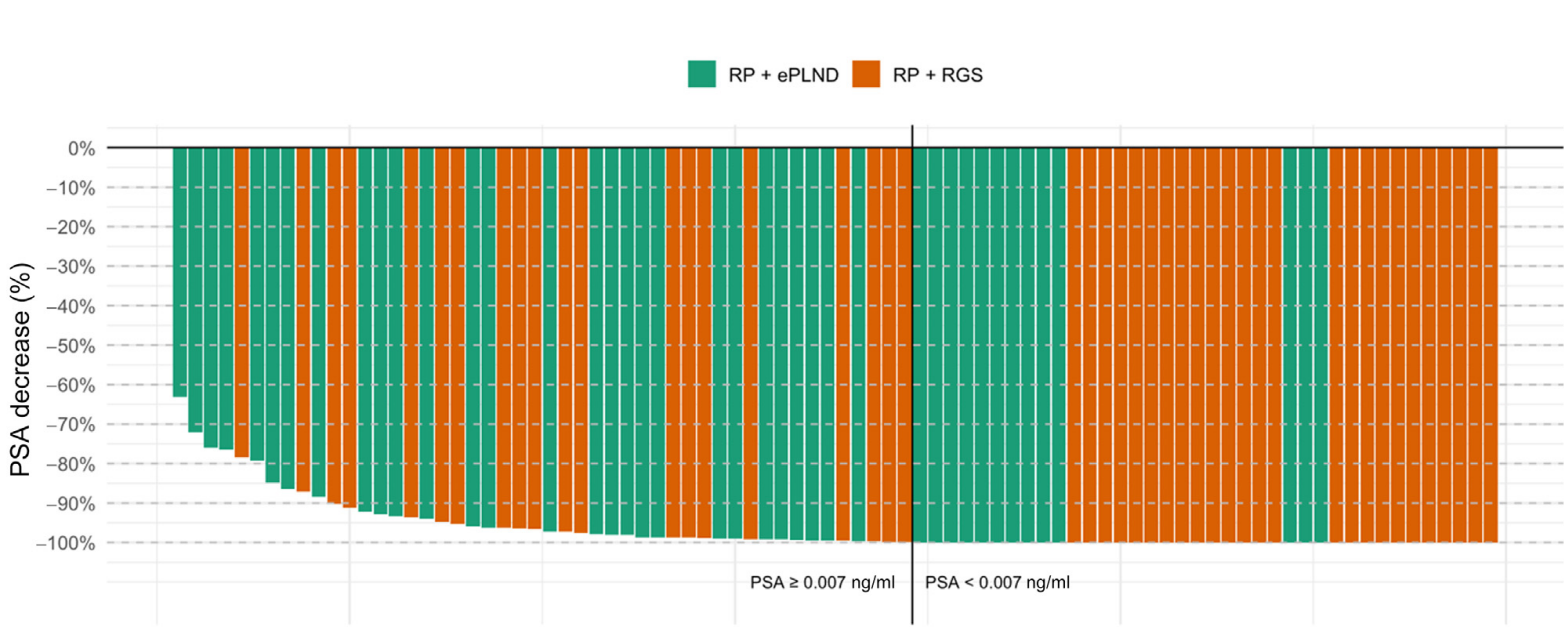
Waterfall plot of the change in prostate-specific antigen (PSA) after radical prostatectomy (RP). ePLND = extended pelvic lymph node dissection; RGS = radioguided surgery.

The median time to PSA measurement after surgery was 9 wk (IQR 8–11) for the RGS group and 10 wk (IQR 9–11) for the conventional ePLND group; the difference was not statistically significant (*p* = 0.5). PSMA-RGS was associated with higher probability of achieving a complete PSA response (<0.07 ng/ml 4–12 wk; odds ratio [OR] 2.46, 95% confidence interval [CI] 1.03–6.06; *p* = 0.045). In total, 25 patients (47%) in the PSMA-RGS group and 13 (30%) in the conventional ePLND group had a complete PSA response. A waterfall plot of the PSA response in cases without additional therapy after RP is shown in Fig. 1.

Median follow-up among patients without BCR was 36.5 mo (IQR 22–47) in the PSMA-RGS group and 75 mo (IQR 72–78) in the conventional ePLND group. At 12 mo, the Kaplan-Meier estimate for the bRFS rate was 39% (95% CI 27–57%) in the PSMA-RGS group and 33% (95% CI 21–51%) in the conventional ePLND group.

Multivariable Cox regression results revealed that a higher number of positive LNs on PET (hazard ratio [HR] 1.35, 95% CI 1.13–1.61) and positive surgical margins (HR 1.76, 95% CI 1.03–3.02) were independent significant predictors of bRFS. PSMA-RGS use was associated with lower probability of bRFS (HR 0.60, 95% CI 0.36–1.01) but the result did not reach statistical significance (*p* = 0.059; Table 2). Median survival times for both groups were adjusted for confounders (positive LNs and surgical margins). The adjusted median bRFS was 5 mo (95% CI 3–9) for the PSMA-RGS group and 2 mo (95% CI 2–6) for the conventional ePLND group (Supplementary Fig. 2).

**Table 2:** Multivariable Cox regression results for biochemical recurrence–free survival

Variable	HR (95% CI)	*p* value
Use of RGS (vs ePLND)	0.60 (0.36–1.01)	0.059
Number of PET positive lymph nodes (continuous variable)	1.35 (1.13–1.61)	**<0.01**
Distribution of lymph nodes (infield only vs out of field)	0.61 (0.33–1.15)	0.13
Surgical margin status (R1 vs R0)	1.76 (1.03–3.02)	**0.04**

CI = confidence interval; ePLND = extended pelvic lymph node dissection; HR = hazard ratio; PET–positron emission tomography; RGS = radioguided surgery.

Median follow-up for TFS was 35 mo (IQR 22–42) for the PSMA-RGS group and 69 mo (IQR 46–75) for the conventional ePLND group. At 12 mo, the Kaplan-Meier estimate for the TFS rate was 48% (95% CI 36–66%) for the RGS group and 23% (95% CI 13–40%) for the conventional ePLND group. In total, further treatment was received by 31 patients in the PSMA-RGS group and 39 patients in the conventional ePLND group. Further treatment in the PSMA-RGS group was a combination of RTx and ADT in 11 patients, ADT alone in 11 patients, RTx in six patients, and repeat PSMA-RGS in three patients. Further treatment in the conventional ePLND group was a combination of RTx and ADT in 17 cases, RTx in 11 cases, ADT in seven cases, and PSMA-RGS in four cases. Multivariable Cox regression analysis revealed that PSMA-RGS use (HR 0.53, 95% CI 0.30–0.94) was associated with lower risk of further treatment, while a higher number of PET-positive LNs (HR 1.29, 95% CI 1.07–1.56) and positive surgical margins (HR 1.86, 95% CI 1.06–3.25) were associated with higher risk of further treatment (Table 3). Median survival times for both groups were adjusted for confounders (positive LNs and surgical margins). The adjusted median TFS was 7 mo (95% CI 5–13) for the PSMA-RGS group and 5 mo (95% CI 4–8) for the conventional ePLND group (Supplementary Fig. 3).

**Table 3:** Multivariable Cox regression results for treatment-free survival

Variable	HR (95% CI)	*p* value
Use of RGS (vs ePLND)	0.53 (0.30–0.94)	**0.03**
Number of PET-positive lymph nodes (continuous variable)	1.29 (1.07–1.56)	**<0.01**
Distribution of lymph nodes (infield only vs out of field)	1.03 (0.55–1.93)	0.9
Surgical margin status (R1 vs R0)	1.86 (1.06–3.25)	**0.03**

CI = confidence interval; ePLND = extended pelvic lymph node dissection; HR = hazard ratio; PET–positron emission tomography; RGS = radioguided surgery.

## 4 Discussion

The emergence of PSMA-RGS as a salvage treatment option for recurrent prostate cancer has shown promising results for LNM detection and potential oncological benefits [7,14]. In the primary setting, different studies with small sample sizes have reported that PSMA-RGS combined with RP is a safe and feasible procedure that can facilitate LNM detection. However, the oncological benefit of adding PSMA-RGS to RP still remains unclear [8,9,15]. To the best of our knowledge, this is the first study to compare RP with PSMA-RGS assistance for PLND versus RP with conventional PLND.

Our data reveal some noteworthy findings. A greater proportion of patients were classified as having pN1 disease after RP with PSMA-RGS (96%) than after conventional ePLND (86%). Furthermore, the median LNM count on histopathology was higher in the PSMA-RGS group (4, IQR 2–5.5) than in the conventional ePLND group (2, IQR 1–3), which could explain the higher probability of a complete PSA response after RP with PSMA-RGS (OR 2.46, 95% CI 1.03–6.06; *p* = 0.045). In addition, PSMA-RGS was associated with longer TFS in comparison to conventional ePLND (HR 0.53, 95% CI 0.30–0.94).

To date, the optimal treatment for patients with clinical evidence of LNM before local treatment remains unclear, with the main controversy regarding whether surgery should be performed [1]. However, if a patient is scheduled for surgery, removal of all tumor lesions seems essential for curative intent, as persistent PSA is an indicator of residual tumor tissue and is associated with poor prognosis for metastasis-free survival and disease-specific survival [16,17]. This is in line with our finding that RGS was associated with longer TFS, despite a higher LNM count on histopathological specimen than in the conventional ePLND group. It is likely that this can be attributed to the surgical method, which facilitates intraoperative LNM detection, but further residual confounding in our observational study cannot be excluded. Cox regression results also showed a trend towards an association with bRFS, but did not reach statistical significance. We believe that this is mostly because of the small sample size for our retrospective analysis; nevertheless, our results show the potential of RGS in this setting.

The median times to BCR (5 mo) and further treatment (6 mo) in our cohort were short, regardless of the use of RGS. This indicates that selection of patients for whom RP combined with PSMA-RGS alone or as part of multimodal treatment is suitable is the most important issue. A key factor is the number of LNMs on preoperative PSMA PET. It is known that a lower LN disease burden is associated with favorable disease-specific outcomes. In two retrospective analyses involving a total of 491 patients who underwent RP with ePLND but without adjuvant treatment, the presence of two or fewer LNMs in the final histopathological specimen was associated with lower risk of BCR [18,19]. These studies were published before PSMA PET/CT was introduced as a routine imaging modality in clinical practice. In a retrospective study of 230 patients, Amiel et al [20] demonstrated that PSMA PET/CT can be used for stratification, as patients with positive LNs on PSMA PET LNs and histopathological pN1 status had the shortest bRFS and TFS, followed by patients with pN1 status but no positive LNs on PSMA PET, and patients without any evidence of LNM (either on histology or on PSMA PET scans). This adds to our previous finding that patients with two or fewer LNMs had longer bRFS, TFS, and metastasis free-survival. The optimal cutoff for the number of positive LNs, especially when using preoperative PSMA PET/CT, is still a matter of debate, as LNMs may not be detected when they are smaller than 3 mm [21]. The ideal cutoff for LNMs is still unknown, especially with newer imaging such as PSMA PET. Our findings add further uncertainty to this topic, as more LNMs were found in histopathological specimens, despite the lack of difference in LNM counts on preoperative PSMA PET scans. RGS contributes to resection of more LNs, which might explain the association with longer TFS. Another key factor is local tumor control. Positive surgical margins were associated with poor prognosis for bRFS and TFS in our cohort, in agreement with the literature [16]. This suggests that patients who have an unresectable primary tumor may not benefit from surgery alone, so adjuvant or early salvage treatment should be discussed as part of a multimodal strategy. New trials are currently evaluating the role of perioperative androgen receptor–targeted therapy or Lu-labeled PSMA radioligands in locally advanced/cN1 prostate cancer, which might also lead to a change in treatment algorithms [22,23].

Limitations of our study include the retrospective design and small sample size. In addition, decisions on salvage treatment and the date of initiation were not made for every patient at our institution, which may lead to bias. Strengths of the study include the compensation for inhomogeneity in patient cohorts via the creation of matched pairs for further analysis, although this may not fully correct for confounding and selection bias. All patients underwent PSA follow-up without adjuvant treatment. Thus, our study represents a real comparison of two surgical approaches, but does not determine the role of surgery in patients with cN1 prostate cancer. Prospective data are necessary to validate these findings.

## 5 Conclusions

RP combined with PSMA-RGS for PLND shows promising results for LNM detection and may facilitate full resection of LNM lesions. This is associated with a higher probability of achieving a complete PSA response after RP and longer TFS. Further investigations, including prospective trials, are necessary to justify a change in clinical practice.

  ***Author contributions***: Matthias M. Heck had full access to all the data in the study and takes responsibility for the integrity of the data and the accuracy of the data analysis.

  *Study concept and design*: Korn, Heck.

*Acquisition of data*: Korn, Lischewski, Staehler.

*Analysis and interpretation of data*: Korn, Heck.

*Drafting of the manuscript*: Korn, Heck.

*Critical revision of the manuscript for important intellectual content*: All authors.

*Statistical analysis*: Korn, Heck.

*Obtaining funding*: None.

*Administrative, technical, or material support*: Heck, Gschwend.

*Supervision*: Heck.

*Other*: None.

  ***Financial disclosures:*** Matthias M. Heck certifies that all conflicts of interest, including specific financial interests and relationships and affiliations relevant to the subject matter or materials discussed in the manuscript (eg, employment/affiliation, grants or funding, consultancies, honoraria, stock ownership or options, expert testimony, royalties, or patents filed, received, or pending), are the following: None.

  ***Funding/Support and role of the sponsor*:** None.

## References

[1] Cornford P., Tilki D., van den Bergh R.C.N. (2025).

[2] Iagaru A., Suarez J.F., Behr S. (2025). Imaging efficacy of [^18^F]CTT1057 PET for the detection of PSMA-positive tumors using histopathology as standard of truth: results from the GuideView phase 2/3 prospective multicenter study. J Nucl Med.

[3] Pienta K.J., Gorin M.A., Rowe S.P. (2021). A phase 2/3 prospective multicenter study of the diagnostic accuracy of prostate specific membrane antigen PET/CT with ^18^F-DCFPyL in prostate cancer patients (OSPREY). J Urol.

[4] Surasi D.S., Eiber M., Maurer T. (2023). Diagnostic performance and safety of positron emission tomography with ^18^F-rhPSMA-7.3 in patients with newly diagnosed unfavourable intermediate- to very-high-risk prostate cancer: results from a phase 3, prospective, multicentre study (LIGHTHOUSE). Eur Urol.

[5] Maurer T., Weirich G., Schottelius M. (2015). Prostate-specific membrane antigen-radioguided surgery for metastatic lymph nodes in prostate cancer. Eur Urol.

[6] Ploussard G., Gandaglia G., Borgmann H. (2019). Salvage lymph node dissection for nodal recurrent prostate cancer: a systematic review. Eur Urol.

[7] Knipper S., Lischewski F., Koehler D. (2025). Biochemical response of <0.1 ng/ml predicts therapy-free survival of prostate cancer patients following prostate-specific membrane antigen-targeted salvage surgery. Eur Urol Oncol.

[8] Gandaglia G., Mazzone E., Stabile A. (2022). Prostate-specific membrane antigen radioguided surgery to detect nodal metastases in primary prostate cancer patients undergoing robot-assisted radical prostatectomy and extended pelvic lymph node dissection: results of a planned interim analysis of a prospective phase 2 study. Eur Urol.

[9] Schilham M.G.M., Somford D.M., Küsters-Vandevelde H.V.N. (2024). Prostate-specific membrane antigen-targeted radioguided pelvic lymph node dissection in newly diagnosed prostate cancer patients with a suspicion of locoregional lymph node metastases: the DETECT trial. J Nucl Med.

[10] Gondoputro W., Scheltema M.J., Blazevski A. (2022). Robot-assisted prostate-specific membrane antigen–radioguided surgery in primary diagnosed prostate cancer. J Nucl Med.

[11] Lunger L., Steinhelfer L., Korn P. (2023). Prostate-specific membrane antigen-radioguided surgery facilitates pelvic lymph node dissection during radical prostatectomy for the treatment of locally advanced prostate cancer with regional lymph node metastases. Eur Urol Oncol.

[12] Quarta L., Mazzone E., Cannoletta D. (2024). Defining the optimal target-to-background ratio to identify positive lymph nodes in prostate cancer patients undergoing robot-assisted [^99m^Tc]Tc-PSMA radioguided surgery: updated results and ad interim analyses of a prospective phase II study. Eur J Nucl Med Mol Imaging.

[13] Greifer N. MatchIt: getting started. The Comprehensive R Network; 2025. https://cran.r-project.org/web/packages/MatchIt/vignettes/MatchIt.html.

[14] Knipper S., Mehdi Irai M., Simon R. (2023). Cohort study of oligorecurrent prostate cancer patients: oncological outcomes of patients treated with salvage lymph node dissection via prostate-specific membrane antigen–radioguided surgery. Eur Urol.

[15] Yılmaz B., Şahin S., Ergül N. (2022). ^99m^Tc-PSMA targeted robot-assisted radioguided surgery during radical prostatectomy and extended lymph node dissection of prostate cancer patients. Ann Nucl Med.

[16] Preisser F., Chun F.K.H., Pompe R.S. (2019). Persistent prostate-specific antigen after radical prostatectomy and its impact on oncologic outcomes. Eur Urol.

[17] Spratt D.E., Dai D.L.Y., Den R.B. (2018). Performance of a prostate cancer genomic classifier in predicting metastasis in men with prostate-specific antigen persistence postprostatectomy. Eur Urol.

[18] Touijer K.A., Mazzola C.R., Sjoberg D.D., Scardino P.T., Eastham J.A. (2014). Long-term outcomes of patients with lymph node metastasis treated with radical prostatectomy without adjuvant androgen-deprivation therapy. Eur Urol.

[19] Schumacher M.C., Burkhard F.C., Thalmann G.N., Fleischmann A., Studer U.E. (2008). Good outcome for patients with few lymph node metastases after radical retropubic prostatectomy. Eur Urol.

[20] Amiel T., Würnschimmel C., Heck M. (2021). Regional lymph node metastasis on prostate specific membrane antigen positron emission tomography correlates with decreased biochemical recurrence-free and therapy-free survival after radical prostatectomy: a retrospective single-center single-arm observational study. J Urol.

[21] Duin J.J., de Barros H.A., Donswijk M.L. (2023). The diagnostic value of the sentinel node procedure to detect occult lymph node metastases in PSMA PET/CT node–negative prostate cancer patients. J Nucl Med.

[22] Ravi P., Kwak L., Xie W. (2022). Neoadjuvant novel hormonal therapy followed by prostatectomy versus up-front prostatectomy for high-risk prostate cancer: a comparative analysis. J Urol.

[23] Eapen R.S., Buteau J.P., Jackson P. (2024). Administering [^177^Lu]Lu-PSMA-617 prior to radical prostatectomy in men with high-risk localised prostate cancer (LuTectomy): a single-centre, single-arm, phase 1/2 study. Eur Urol.

