# Supplementary material for: Radical Prostatectomy Combined with Prostate Specific Membrane Antigen–radioguided Lymph Node Dissection is Associated with Longer Treatment-free Survival for Patients with Primary Lymph Node–positive Prostate Cancer
Source: Eur Urol Open Sci. 2025 Nov 14;82:201–7. doi: 10.1016/j.euros.2025.10.018 (PMC12664431; doi:10.1016/j.euros.2025.10.018)
Supplement: Supplementary Data 1 [file mmc1.docx]

**Supplementary**

**
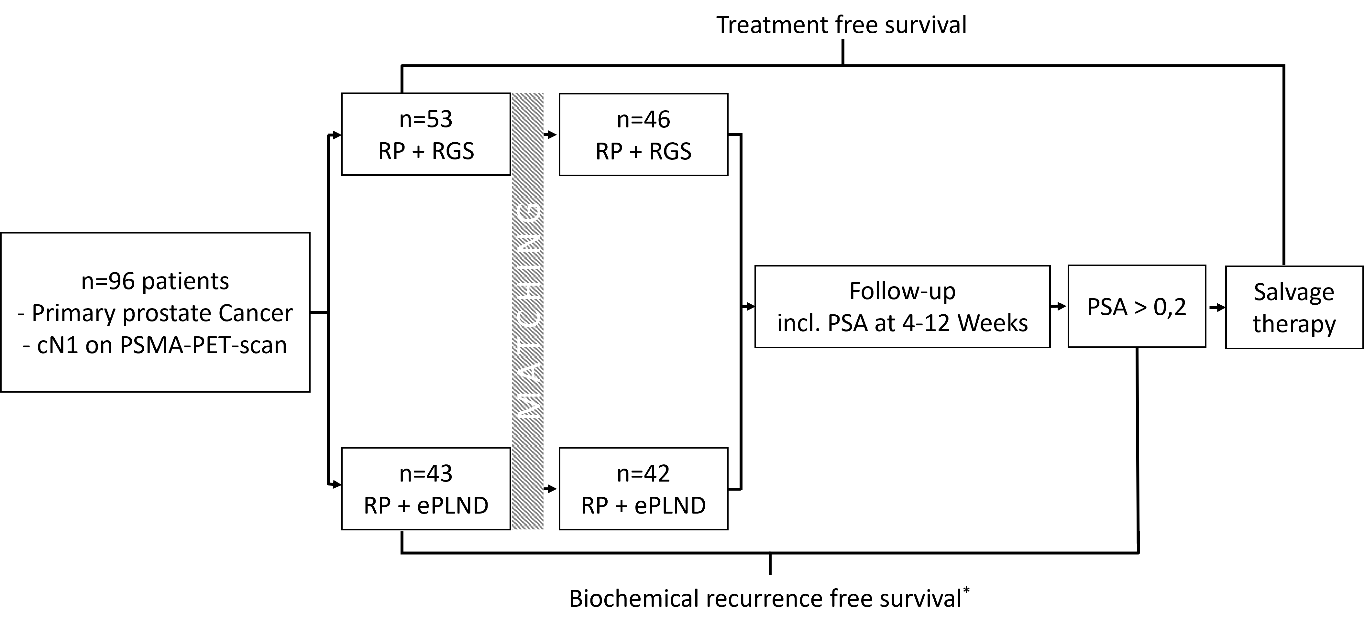
**

*when treatment was initiated before PSA reached 0.2ng/ml, date of treatment initiation was defined as BCR
Supplementary figure 1: Flowchart depicting the worksteps


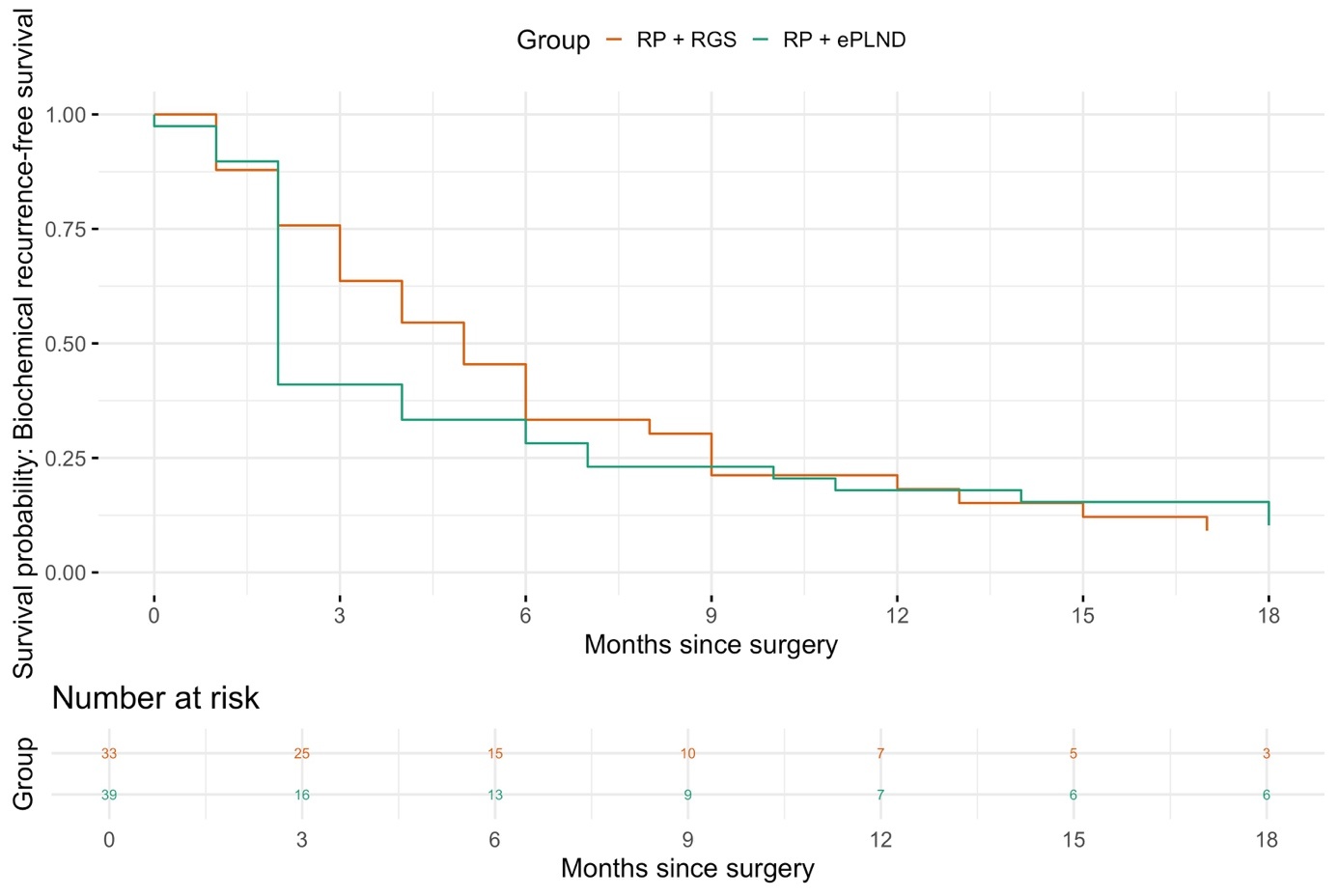


Supplementary figure 2: Adjusted survival curve bRFS


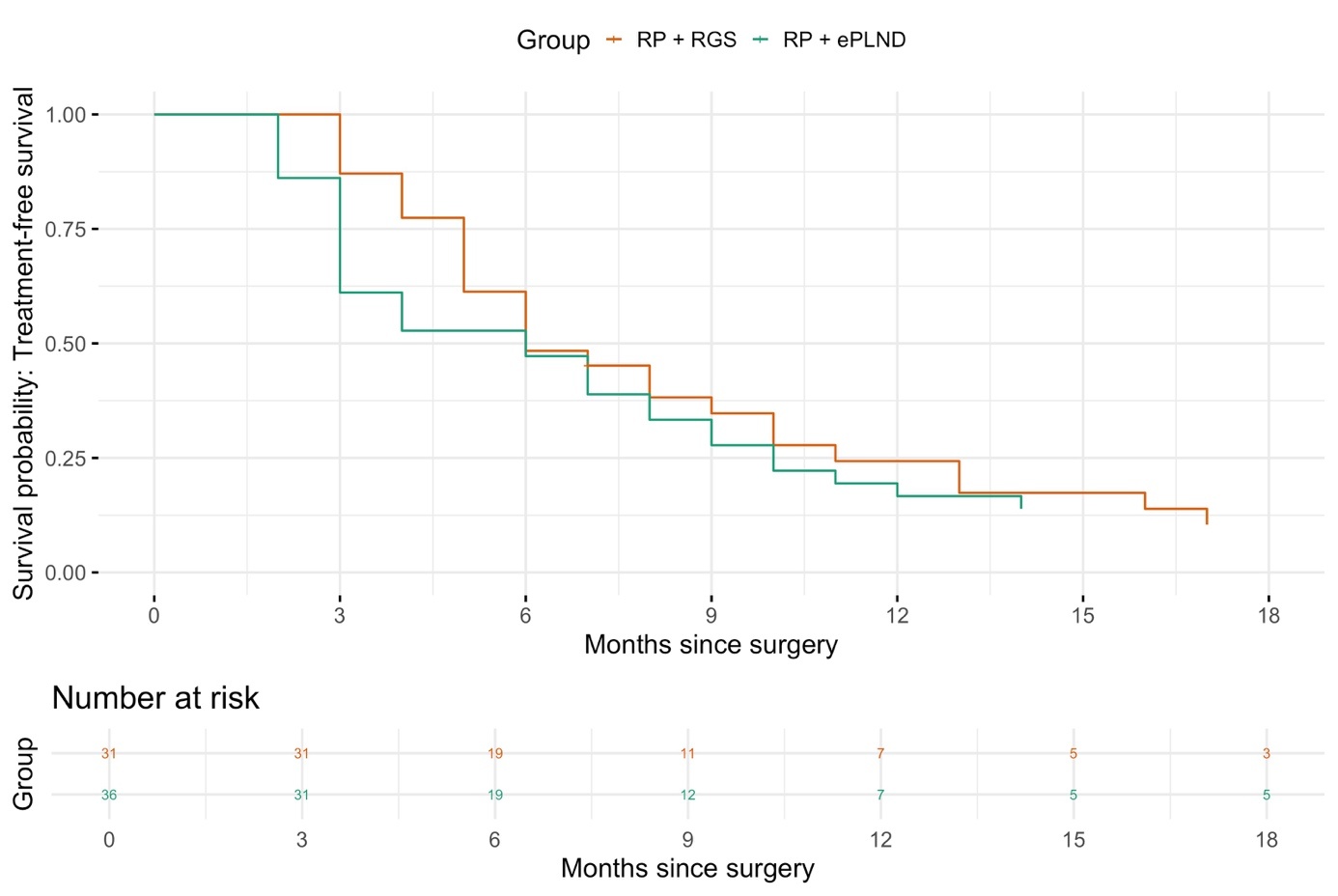


Supplementary figure 3: Adjusted survival curve TFS
